
JOURNAL INFORMATION
==============================
NLM Title Abbreviation: ChemistryOpen
Journal ID: ChemistryOpen
NLM Journal ID: 101594811
Journal ID: open

ChemistryOpen

EISSN: 2191-1363
Publisher: Wiley

ARTICLE INFORMATION
==============================
PMCID: PMC3892190
DOI: 10.1002/open.201380521
Article version: 1
Subjects: Masthead

Masthead: ChemistryOpen 5-6/2013

Print publication date: 2013 Dec
Electronic publication date: 2013 Dec 19
Volume: 2
Issue: 5-6
First page: 168
Last page: 168
Copyright: Copyright © 2013 WILEY-VCH Verlag GmbH & Co. KGaA, Weinheim
Copyright year: 2013
License: Re-use of this article is permitted in accordance with the Creative Commons Deed, Attribution 2.5, which does not permit commercial exploitation.
License URL: https://creativecommons.org/licenses/by/2.5/

==============================
Austria
Belgium
Czech Republic
France
Germany
Greece
Hungary
Italy
Netherlands
Poland
Portugal
Slovakia
Spain
Sweden
Switzerland

ChemistryOPEN Masthead

ChemPubSoc Europe www.chemistryopen.org

### Editorial Advisory Board

### *Chairmen*

**Ramón Martínez-Máñez (Spain)**
**Thomas Wirth (UK)**

### *Honorary Chairman*

**Jean-Marie Lehn (France)**

### *Members*

Fernando Albericio (Spain)
Valentin Ananikov (Russia)
Didier Astruc (France)
Jochen Autschbach (USA)
Martin Banwell (Australia)
Vincenzo Barone (Italy)
F. Matthias Bickelhaupt (The Netherlands)
Kathrin Breuker (Austria)
Young Keun Chung (South Korea)
Richard G. Compton (UK)
Ulf Diederichsen (Germany)
Lothar Dunsch (Germany)
Antony Fairbanks (New Zealand)
Ernest Giralt (Spain)
Stefan Grimme (Germany)
Dirk Guldi (Germany)
Akira Harada (Japan)
Narayan Hosmane (USA)
Ken Houk (USA)
Osamu Ito (Japan)
Christoph Janiak (Germany)
Kazunori Kataoka (Japan)
Yoshiaki Kiso (Japan)
Mats Larhed (Sweden)
Lechoslav Latos-Grażyński (Poland)
Thisbe Lindhorst (Germany)
Luis M. Liz-Marzán (Spain)
Carlos Lodeiro (Portugal)
Todd L. Lowary (Canada)
Jean Martinez (France)
Pedro Matias (Portugal)
Stefan Matile (Switzerland)
Pedro Molina (Spain)
K. C. Nicolaou (USA)
Berit Olofsson (Sweden)
Oliver Reiser (Germany)
Kari Rissanen (Finland)
Knut Rurack (Germany)
James Rusling (USA)
Albert Schenning (The Netherlands)
Robert Schlögl (Germany)
Patrick Schmuki (Germany)
Ulrich S. Schubert (Germany)
Min Shi (China)
Norio Shibata (Japan)
Rint Sijbesma (The Netherlands)
Choong Eui Song (South Korea)
Petr Štěpnička (Czech Republic)
Brian Stoltz (USA)
Steve Suib (USA)
Reshef Tenne (Israel)
Matthew Todd (Australia)
Claudio Toniolo (Italy)
Jaume Veciana (Spain)
Hans-Achim Wagenknecht (Germany)
Ralph Weissleder (USA)
Gunther Wittstock (Germany)
Vivian W. W. Yam (China)
Chun-Hua Yan (China)
Jincai Zhao (China)

### Open Access and Copyright

All research articles published by *ChemistryOpen* are fully open access: immediately freely available to read, download and share. All *ChemistryOpen* articles are published under the terms of the Creative Commons Attribution Non-Commercial License which permits use, distribution and reproduction in any medium, provided the original work is properly cited and is not used for commercial purposes.

Copyright on any research article published by *ChemistryOpen* is retained by the author(s). Authors grant Wiley a license to publish the article and identify itself as the original publisher. Authors also grant any third party the right to use the article freely as long as its integrity is maintained and its original authors, citation details and publisher are identified.

### Use by commercial "for-profit" organisations

Use of Wiley Open Access articles for commercial, promotional, or marketing purposes requires further explicit permission from Wiley and will be subject to a fee. Copyright 2012 Wiley-VCH Verlag GmbH & Co. KGaA, Weinheim. All rights reserved. Requests to reproduce material from this journal content should be addressed to rights@wiley-vch.de, and reprints of *ChemistryOpen* articles can be purchased from chem-reprints@wiley-vch.de.

### Disclaimer

The Publisher and Editors cannot be held responsible for errors or any consequences arising from the use of information contained in this journal; the views and opinions expressed do not necessarily reflect those of the Publisher and Editors, neither does the publication of advertisements constitute any endorsement by the Publisher and Editors of the products advertised.

Wiley Open Access articles posted to repositories or websites are without warranty from Wiley of any kind, either express or implied, including, but not limited to, warranties of merchantability, fitness for a particular purpose, or non-infringement. To the fullest extent permitted by law Wiley disclaims all liability for any loss or damage arising out of, or in connection with, the use of or inability to use the content.

**Editors:**
Karen Hindson, Haymo Ross

**Deputy Editor:**
Natalia Ortúzar

**Associate Editor:**
Saskia Neubacher

**Secretary:**
Vanessa Müller

**Production:**
Stefanie Klein, Margitta Schmitt

**Telephone:**
(+ 49) 6201-606-305

**Fax:**
(+ 49) 6201-606-203

**E-mail:**
chemistryopen@wiley-vch.de

**Courier Services:**
Boschstrasse 12
69469 Weinheim (Germany)

**Regular Mail:**
Postfach 10 11 61
69451 Weinheim (Germany)

**Homepage:**
http://www.chemistryopen.org

**Manuscript Submission & Personal Homepage**

manuscriptXpress

**http://www.manuscriptxpress.com**

**Customer Services:**
www.wileycustomerhelp.com

**Marketing:**
Monika Silz (ext. 458, fax: ext. 100,
e-mail: msilz@wiley-vch.de)

**Advertising:**
Marion Schulz (ext. 565, fax: ext. 550,
e-mail: mschulz@wiley-vch.de, jspiess@wiley-vch.de)

**Copyright Permissions:**
Bettina Loycke (ext. 280, fax: ext. 447,
e-mail: rights@wiley-vch.de)

**Reprints, e-prints, posters:**
Carmen Leitner (fax: ext. 226)
e-mail: chem-reprints@wiley-vch.de

**Publishers:**
WILEY-VCH Verlag GmbH & Co. KGaA,
Postfach 10 11 61
69451 Weinheim (Germany)
http://www.wiley-vch.de/

**Publication:**
6 issues in 2013

© 2013 WILEY-VCH Verlag GmbH & Co. KGaA, Weinheim.
